# A propos d'un rare cas de tumeur Rhabdoïde Teratoïde atypique du systeme nerveux central chez une femme enceinte

**DOI:** 10.11604/pamj.2015.20.2.5877

**Published:** 2015-01-05

**Authors:** Mohammed Afif, Jihane Khalil, Fadila Kouhen, Abdellah Aissa, Youssef Omour, Mustapha Elkabous, Hanan Elkacemi, Tayeb Kebdani, Noureddine Benjaafar

**Affiliations:** 1 Service de Radiothérapie, Institut National d'Oncologie, Université Mohammed V, Rabat, Maroc; 2 Service de Radiologie, Institut National d'Oncologie de Rabat, Université Mohammed V, Rabat, Maroc; 3 Service d'Oncologie Médicale, Institut National d'Oncologie, Université Mohammed V, Rabat, Maroc

**Keywords:** Tumeur rhabdoide tératoide atypique, cervelet, femme enceinte, atypical rhabdoid tumor, cerebellum, pregnant woman

## Abstract

Les tumeurs rhabdoïdes tératoïdes atypiques du système nerveux central sont des tumeurs pédiatriques rares et de mauvais pronostic. La littérature rapporte une dizaine de cas chez l'adulte dont deux survenus au cours d'une grossesse. Nous rapportons dans ce travail, le cas d'une femme de 25 ans, enceinte de 14 semaines d'aménorrhée, qui a été opérée pour une tumeur rhabdoïde tératoïde atypique de la fosse cérébrale postérieure. Un complément thérapeutique a été discuté chez la patiente après interruption thérapeutique de grossesse, mais la patiente fut décédée avant de démarrer le traitement adjuvant. Nous décrivons brièvement les caractéristiques des tumeurs rhabdoïdes, et les particularités de sa prise en charge chez l'adulte.

## Introduction

Les tumeurs rhabdoïdes dites également tératoïdes atypiques (TRTA) du système nerveux central (SNC) sont des tumeurs pédiatriques rares et agressives. Les cas rapportés chez l'adulte sont rares [1], et les cas décrits chez la femme enceinte sont exceptionnels [2, 3]. Ainsi, notre cas représente le 3^ème^ cas décrit chez une femme enceinte.

## Patient et observation

**Figure 1:**
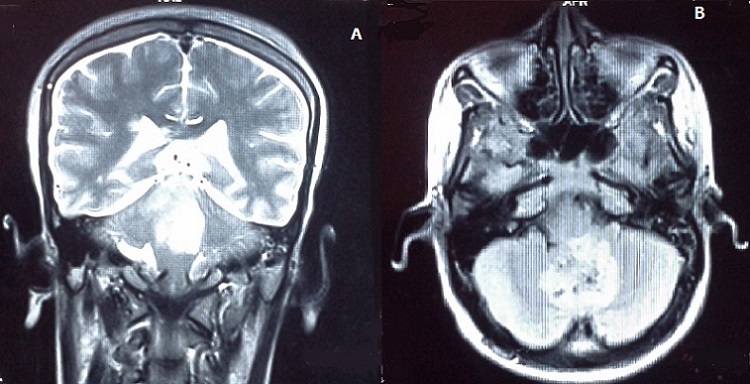
Images IRM en coupe frontale «A» et sagitale «B», objectivant une tumeur siégeant au niveau du vermix, avec un hypersignal en T2

**Figure 2:**
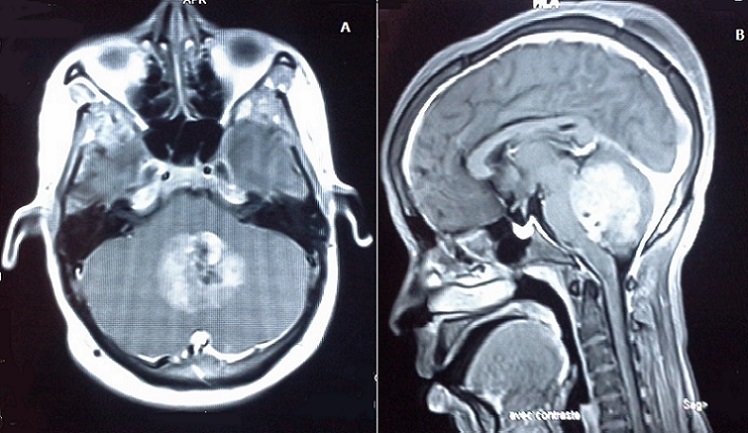
Images IRM T1 après injection de Gadolinium (coupe axiale «A» et sagittale «B»), objectivant la tumeur du vermis avec une forte prise de contraste

Il s'agit d'une femme de 25 ans, sans antécédents pathologiques notables, primipare et enceinte de 14 semaines d'aménorrhée, qui a présenté 3 semaines avant le diagnostic de la tumeur, un syndrome d'hypertension intracrânienne associé à une baisse de l'acuité visuelle bilatérale, et un syndrome cérébelleux. Une imagerie par résonnance magnétique (IRM) cérébrale a été réalisée en urgence, et avait objectivé un processus sous tentoriel, de siège médian, occupant le vermix, infiltrant les hémisphères cérébelleux latéralement, et occupant le V4 avec refoulement du tronc cérébral, ce processus mesurant 56x46x40 mm, est tissulaire, hétérogène, en hyposignal en T1, hyper signal en T2, et prend fortement le contraste en T1 après injection du gadolinium (Figure 1 et Figure 2). Il est associé à une hydrocéphalie, et un engagement amygdalien. La patiente a bénéficié initialement en urgence d'une dérivation ventriculopéritonéale, puis secondairement d'une exérèse large et fragmentée de la tumeur.

**Figure 3:**
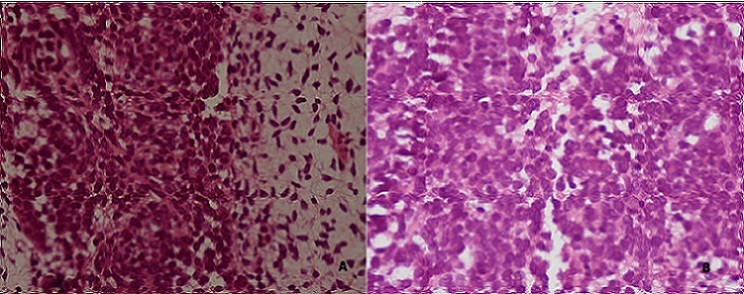
Aspect anatomopathologique de la tumeur: prolifération tumorale d'architecture polymorphe «A»; faite de cellules rhabdoïdes rondes, à cytoplasme éosinophile, comportant des inclusions hyalines intracytoplasmiques «B»

L’étude anatomopathologique avait objectivé un processus tumoral d'architecture polymorphe, constitué de nappes cellulaires blastémateuses, faites de cellules indifférenciées à noyaux hyperchromatiques excentrés et comportant des inclusions intracytoplasmiques, ces nappes étaient séparées par un tissu mésenchymateux très dense comportant des cellules rhabdomyoblastiques regroupées en amas. L'aspect était en faveur d'une tumeur rhabdoïde tératoïde atypique, grade IV de l'OMS (Figure 3). L'immunohistochimie a confirmé le diagnostic avec une positivité de la vimentine; EMA, et la synaptophysine. La patiente a été adressée au service de radiothérapie pour une radiothérapie adjuvante, les options thérapeutiques ont été discutées avec la famille, et une interruption thérapeutique de grossesse à été indiquée, mais la patiente fut décédée dans les suites opératoires, par détresse respiratoire, trois semaines après la chirurgie, et avant de démarrer le traitement adjuvant.

## Discussion

Les TRTA du SNC constituent une entité pathologique rare, survenant principalement chez l'enfant, avec une fréquence ne dépassant pas 1 à 2% des tumeurs cérébrales pédiatriques [4]. Les cas décrits chez l'adulte sont très rares, et seulement deux cas de TRTA chez une femme enceinte ont été publiés jusqu’à nos jours [2, 3]. Décrites pour la 1^ère^ fois en 1978 par Beckwith et Palmer comme une tumeur primitive rénale extrêmement agressive [5], le 1^er^ cas de tumeur rhabdoïde maligne cérébrale a été diagnostiqué chez un nourrisson âgé de trois mois par Briner (1985) [6]. La symptomatologie clinique des TRTA n'est pas spécifique, et varie en fonction de l’âge, et du siège de la tumeur (Syndrome d'HTIC, crises convulsives, syndrome cérébelleux ou pyramidal…). La symptomatologie de l'adulte peut être plus accentuée, et amène à un diagnostic plus précoce [1, 7]. L'IRM est l'examen clé au diagnostic notamment chez la femme enceinte, à l'IRM, les tumeurs apparaissent isointenses par rapport au cortex en T2; associées à des calcifications, et des remaniements hémorragiques et kystiques, cet aspect peut poser un problème de diagnostic différentiel avec les médulloblastomes, ce qui était le cas pour notre patiente [8]. Le diagnostic de certitude est histologique, il s'agit d'une prolifération cellulaire diffuse, faite de cellules rhabdoïdes typiques polygonales ou rondes, à cytoplasme abondant, ces cellules sont siège d'inclusions éosinophiles hyalines à bordure bien définie, refoulant un noyau excentré vésiculeux, avec nucléole proéminent, et montrant des atypies marquées. Dans 85% des cas, le contingent rhabdoïde s'associe à d'autres éléments tissulaires.

L'immunohistochimie présente un grand intérêt pour la confirmation diagnostique, les TRTA expriment de la vimentine, l'EMA, et de la cytokératine, elles présentent une positivité variable pour la GFAP, la NSE, la protéine S100 et la Synaptophysine [9, 10]. Sur le plan moléculaire, les TRTA sont associées à une délétion du gène hSNF5/INI1 (gène suppresseur de tumeur), situé au niveau du chromosome 22q11 [11, 12], cette mutation du gène hSNF5/INI1 est pathognomonique des tumeurs avec composante rhabdoïde, et pourra être demandée dans les cas de difficultés diagnostiques [12]. L'exérèse chirurgicale la plus complète semble être le seul traitement ayant permis des survies prolongées. La radiothérapie cérébrospinale adjuvante est recommandée à cause des disséminations méningées fréquentes [1, 13]. La dose recommandée est de 36 Gy sur l'axe cérébrospinal, avec une surimpression de 24 Gy sur le lit tumoral. Certains auteurs recommandent une désescalade des doses à moins de 50 Gy, afin de réduire la toxicité neurologique, sans différence sur la survie globale et la survie sans récidive. La chimiothérapie intrathécale à base de cytarabine a également permis une amélioration clinique [14]. Dans les deux cas de TRTA chez une femme enceinte publiés dans la littérature, les patientes avaient une grossesse assez évoluée, l'une à 33 SA, et la 2^ème^ à 36 SA, elles ont été opérées pendant la grossesse, et elles ont pu bénéficier de radiothérapie adjuvante après extraction du fœtus [2, 3]. Dans notre cas, la patiente était encore au 1^er^ trimestre de grossesse (14 SA), et une interruption thérapeutique s'avérait nécessaire avant toute décision d'un traitement adjuvant. Le pronostic des TRTA reste mauvais, avec une médiane de survie dans les séries publiées ne dépassant pas les 8 mois [1], ceci est du au caractère agressif de la tumeur, et son potentiel métastatique élevé. Le pronostic chez l'adulte semble plus mauvais, mais la rareté des cas ne permet pas de confirmer l'impact de l’âge sur le pronostic.

## Conclusion

L'observation que nous avons décrit confirme le caractère agressif des tumeurs rhabdoïdes tératoïdes atypiques de l'adulte, et l'association avec la grossesse dans notre contexte a posé des difficultés pour continuer le traitement adjuvant. Une prise en charge multidisciplinaire impliquant neurochirurgien, oncologue radiothérapeute, et obstétricien est indispensable afin de mieux gérer des cas similaires.
